# Supplementary material for: Arginine Decarboxylase Is Essential for Pneumococcal Stress Responses
Source: Pathogens. 2021 Mar 2;10(3):286. doi: 10.3390/pathogens10030286 (PMC7998104; doi:10.3390/pathogens10030286)
Supplement: Supplementary file 1 [file pathogens-10-00286-s001.zip › supplementary -final/Supplementary Table S2.docx]

Table S2. Sequences of primers used in this study

| **Primer** | **Sequence (5'→3')** |
| --- | --- |
| *speE For* | TGCGGATGATTTCGTCTACAATG |
| *speE Rev* | GCTTAGTCCTGGTCGCAATC |
| *aguA For* | CTGGGGATCATTTTCGTCAT |
| *aguA Rev* | CTGGGGATCATTTTCGTCAT |
| *nspC For* | ATGTATTTGCGCCTGCTTTC |
| *nspC Rev* | TGGTGCACAAGGGTCATAGA |
| *noxA For* | CAATAAACTTTCTGACAAGAGCCAAC |
| *noxA Rev* | ATATCAACAAGGACAACTTCTTTTCC |
| *tpx For* | CGACAAGGCGCTTGATTTTT |
| *tpx Rev* | TTGAGTTGAGCAGATGCCTG |
| *psaA For* | ATGCCAAGAAAACTGAAAACAAAGAC |
| *psaA Rev* | CAAGGTTAAGCCAAGCGTGTG |
| *htrA For* | CTATTGCCATCGGTAGCCCGTTAG |
| *htrA Rev* | GTTACCTGGGTTAATAGCAGTATC |
| *prtA For* | GAGTCAGTGGAAGAAATGGAAGC |
| *prtA Rev* | TTATCAAGGATTGGTTCGTGGTTC |
| *gor For* | CGTAAATGGTGAACTGATTCGTG |
| *gor Rev* | TTCTTCCCAGGCAAATACATCATC |
| *gloA For* | ACCATGCCTTCACGATTGTC |
| *gloA Rev* | ACCCATCACCAACCACATAC |
| *SP_0313 For* | CGCTATCAAGATCAGGGCTT |
| *SP_0313 Rev* | GGCTACAGAAGGCGTTGATT |
| *SP_1550 For* | GCATATCAATTACCGACCGTATGG |
| *SP_1550 Rev* | GTTCTCCTTTTGGCAAGTTTTGTTC |
| *fhuD For* | GCGCTTGTTCTTCTAATTCTGT |
| *fhuD Rev* | CAGGCGCATGCTCTTTAGAA |
| *piaA For* | TTCGTAGACAAATTCAAAGAAATCGC |
| *piaA Rev* | ATTAGCCTTGGTAGAAGTCCAGTAG |
| *Rgg For* | TGCTCTACCTCTCAGTTATCTCG |
| *Rgg Rev* | TGTTATCCAAAATCTCAAAGAAACGG |
| *codY For* | ATGGCGGTCAATACCCTTTC |
| *codY R ev* | CCTTCATTCCAAGTGAGCGA |
| *tcs04 Fev2* | CACTGACTAGAGAGCGTCTTTTG |
| *tcs04 Rev2* | TTCCCTCAACTTACCAATATGAACG |
| *spxR For* | AAGATTATTTGGACTTGGTTCGTAAG |
| *spxR Rev* | AATTGTCGTGCTTGGTGATTTATC |
| *psaR For* | CCCTGCCGTAACTGAAATGA |
| *psaR Rev* | AGAGACCAGTTTGAGACCGA |
| *pcpA For* | TTCTTAATATGAATTTCCTTTAATG |
| *pcpA Rev* | GGTAATAATATCAACTCATTGC |
| *ciaH For* | TCGGTGTCTTCACCCTGATT |
| *ciaH Rev* | GAAGCTTATCGTCCACCGAA |
| *etrx2 For* | CTCAAGCAGCACAACAGCCAAAAC |
| *etrx2 Rev* | GGTTTCGCCGCTAGTTCCATCAAC |
| *etrx1 For* | TGATTACAAGGGCAAGAAAGTCTATC |
| *etrx1 Rev* | CTTTAGCAATCTCATCCGTATCTGG |
| *clpP For* | GAACAAACAAGCCGTGGAGAACG |
| *clpP Rev* | CCACCTGGTGTATTGACATAAAGG |
