# Supplementary figures and images for: Arginine Decarboxylase Is Essential for Pneumococcal Stress Responses
Source: Pathogens. 2021 Mar 2;10(3):286. doi: 10.3390/pathogens10030286 (PMC7998104; doi:10.3390/pathogens10030286)

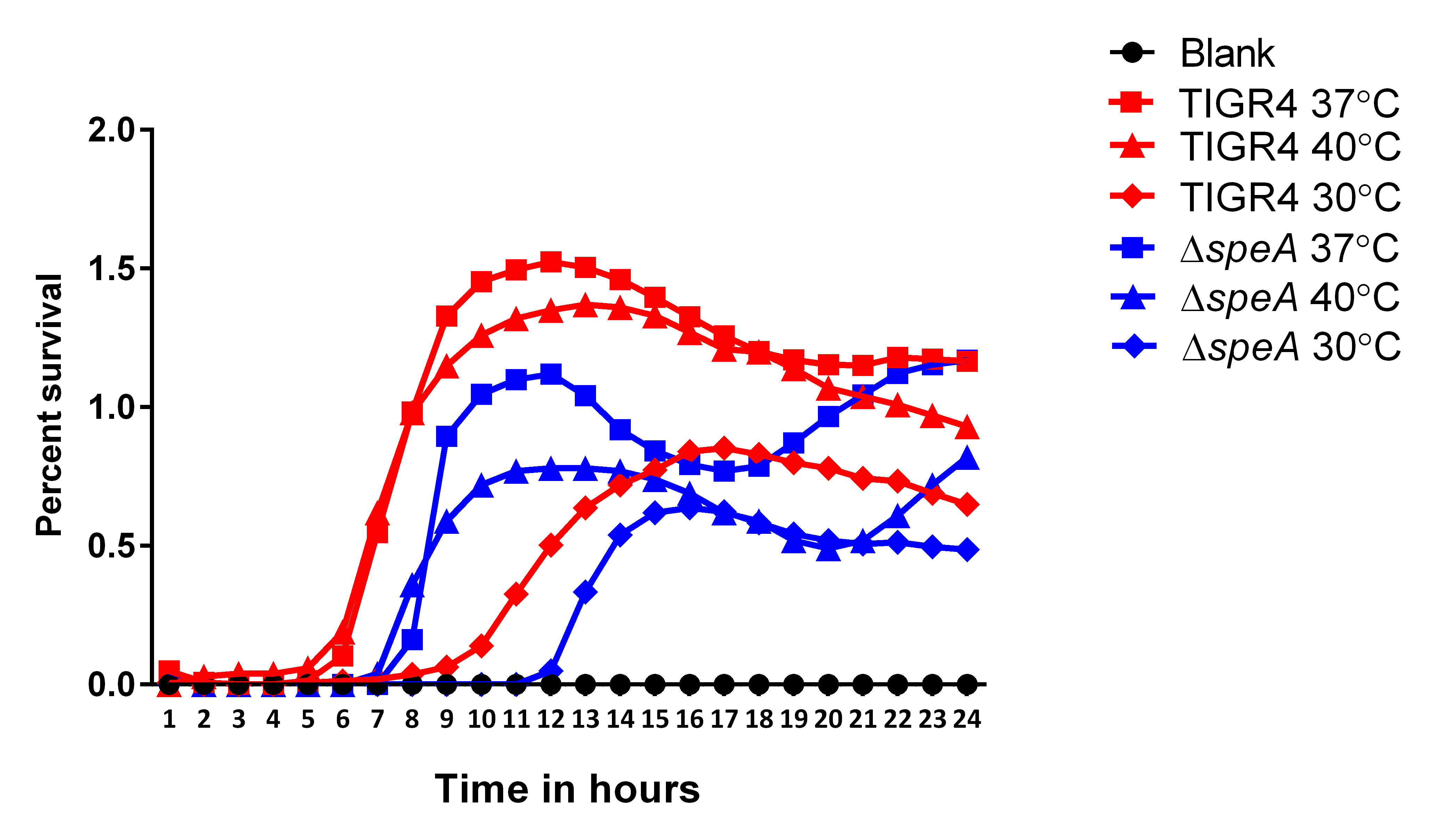

Supplement: Supplementary file 1 [file pathogens-10-00286-s001.zip › supplementary -final/pathogens-1082799 - figure S1.jpg]
