# Supplementary material for: Arginine Decarboxylase Is Essential for Pneumococcal Stress Responses
Source: Pathogens. 2021 Mar 2;10(3):286. doi: 10.3390/pathogens10030286 (PMC7998104; doi:10.3390/pathogens10030286)
Supplement: Supplementary file 1 [file pathogens-10-00286-s001.zip › supplementary -final/Supplementary Table S1.doc]

Table S1. Changes in gene expression between *ΔspeA* and TIGR4 cultured presence or absence of hydrogen peroxide that are < 2.0 folds.

| **Gene** | **Description** | ***ΔspeA*/TIGR4 Fold change Untreated** | ***ΔspeA*/TIGR4 Fold change 2.5 mM H_2_O_2_** |
| --- | --- | --- | --- |
| *spxB* | Pyruvate oxidase | *-1.3* | *-1.0* |
| *ahpD* | Alkyl hydroperoxide reductase | -1.6 | 1.6 |
| *rgg* | Transcriptional regulator | *1.0* | *1.8* |
| *codY* | GTP-sensing transcriptional pleiotropic repressor | 1.9 | *1.3* |
| *spxR* | Hypothetical protein | -1.4 | 1.2 |
| *tcs04* | Response regulator | *-1.3* | -1.3 |
| *psaR* | Iron-dependent transcriptional regulator | -1.6 | *1.3* |
| *ciaH* | Sensor histidine kinase |  | -1.6 |
| *etrx1* | Thioredoxin family protein | *-1.0* | 1.8 |
| *etrx2* | Thioredoxin family protein | -1.3 | *1.2* |
| *potD* | Putrescine ABC transporter |  | *1.1* |
| *pcpA* | Choline binding protein | 1.8 | *1.0* |
| *clpP* | ATP-dependent protease proteolytic | *1.2* | *1.7* |
| *tpxD* | Thiol peroxidase | *1.0* |  |
| *gor* | Glutathione reductase | *1.2* | *-1.2* |
| *gloA* | Lactoylglutathione lyase | -1.9 | 1.5 |
| ***SP_0313* | Glutathione peroxidase | 1.2 | *-1.3* |
| ***SP_1550* | Glutathione S-transferase | *-1.1* | *-1.1* |
| *psaA* | Manganese ABC transporter substrate-binding lipoprotein | 1.4 |  |
| *psaB* | Manganese ABC transporter | *-1.1* | *1.2* |
| *piaA* | Iron-compound ABC transporter, iron-compound-binding protein | *1.2* | 1.9 |
| *fhuD* | Iron-compound ABC transporter | *-1.2* |  |
| *ciaR* | Cytoplasmic response regulator | *-1.1* | -1.6 |
| *aguA* | Putative agmatine deiminase | *-1.1* |  |

**: Locus tag; Fold changes in *italics* are not significant
